# Optimal Evidence in Difficult Settings: Improving Health Interventions and Decision Making in Disasters

**DOI:** 10.1371/journal.pmed.1001632

**Published:** 2014-04-22

**Authors:** Martin Gerdin, Mike Clarke, Claire Allen, Bonnix Kayabu, William Summerskill, Declan Devane, Malcolm MacLachlan, Paul Spiegel, Anjan Ghosh, Rony Zachariah, Saurabh Gupta, Virginia Barbour, Virginia Murray, Johan von Schreeb

**Affiliations:** 1 Centre for Research on Health Care in Disasters, Health Systems and Policy, Department of Public Health Sciences, Karolinska Institutet, Stockholm, Sweden; 2 All-Ireland Hub for Trials Methodology Research, Centre for Public Health, Institute of Clinical Sciences, Queens University Belfast, Royal Hospitals, Belfast, United Kingdom; 3 Evidence Aid, Oxford, United Kingdom; 4 Evidence Aid, Centre for Global Health & School of Psychology, Trinity College Dublin, Dublin, Ireland; 5 The Lancet, London, United Kingdom; 6 National University of Ireland Galway & West Northwest Hospitals Group, Ireland; 7 Centre for Global Health & School of Psychology, Trinity College Dublin, Dublin, Ireland; 8 United Nations High Commissioner for Refugees, Geneva, Switzerland; 9 Extreme Events and Health Protection, Public Health England, London, United Kingdom; 10 Operational Research, Médecins Sans Frontières- Brussels Operational Centre, Gasperich, Luxembourg; 11 Department of Immunisation, Hepatitis and Blood Safety, Public Health England, London, United Kingdom; 12 Medicine Editorial Director, PLOS, Brisbane, Australia

## Abstract

Martin Gerdin and colleagues argue that disaster health interventions and decision-making can benefit from an evidence-based approach

*Please see later in the article for the Editors' Summary*

**Summary Points**

- As for any type of health care, decisions about interventions in the context of natural disasters, conflict, and other major healthcare emergencies must be guided by the best possible evidence.
- Disaster health interventions and decision making can benefit from an evidence-based approach.
- We outline how systematic reviews and methodologically sound research can build a much-needed evidence base.
- We do this from the standpoint of Evidence Aid, an initiative that aims to improve access to evidence on the effects of interventions, actions, and policies before, during, and after disasters and other humanitarian emergencies, so as to improve health-related outcomes.

## Introduction

Natural and man-made disasters are a global concern, with the potential to displace, kill, and injure large numbers of people, disrupt health systems, devastate food, water, and energy supplies, shatter economies, and cause massive destruction of infrastructure [1]. Recent major disasters include the Haiti earthquake (2010), the tsunami and radiation leaks in Japan (2011), Superstorm Sandy affecting North America (2012), and typhoon Haiyan in the Philippines (2013). The chronic fragile situation in countries such as Afghanistan over the last few decades and, more recently, the conflict in Syria can be considered man-made disasters. There are many less high-profile disasters, such as landslides in Uganda, mudslides in Bolivia, and floods in Burkina Faso. Disasters pose serious threats to health, and the lack of evidence base in disaster health response has been internationally recognised, for example after the 2010 Haiti earthquake [2].

Even if it is not possible to predict the specifics of disasters, they happen regularly and can be prepared for. The level of evidence in the disaster health response should be the same as for other health settings. A needs assessment survey by Evidence Aid (Box 1) gathered information on the views and attitudes towards systematic reviews of people involved in planning for, and responding to, disasters [3]. It showed that research evidence could play a central role in improving the effectiveness of international assistance in the planning, delivery, and recovery phases of a disaster [4]. In this paper, we discuss how disaster health interventions and decision making can benefit from an evidence-based approach, similar to other healthcare settings, and outline how methodologically sound research can build a much-needed evidence base (Box 2, Box 3, Box 4).

**Box 1.** Outline of the Evidence Aid initiative

Evidence Aid (www.evidenceaid.org) is an initiative that tries to improve the quality of evidence and seeks to identify which, if any, systematic reviews from the thousands available in *The Cochrane Database of Systematic Reviews* and elsewhere are relevant to the disaster context, and which unanswered questions should be tackled in new reviews. The aim of Evidence Aid is to improve access to evidence on how to intervene and the eventual effects before, during, and after natural disasters and other humanitarian emergencies, so as to improve health-related outcomes.

**Box 2.** Do electric fans reduce adverse health effects during heat waves?

Since 2000, an estimated 150,000 people have died in heat waves across the world. The frequency and severity of heat waves are expected to increase in the future. Electric fans have been available for decades, and are widely used globally. A recent Cochrane Review sought to determine how their use affects important health outcomes during heat waves [26]. The review revealed substantial gaps in research in the international published and unpublished literature about the use of electric fans during heat waves, and was unable to provide robust guidance to health policy makers in support of electric fans. Instead, it recommends the conduct of randomised trials and includes the design of a trial to assess the effects of electric fans on health outcomes during heat waves. This first Evidence Aid review is an example of a systematic review that highlights knowledge gaps in important disaster health areas and might provide a basis for methodologically sound research, be it randomised or observational.

**Box 3.** What are the effects of interventions to improve the microbial quality of drinking water for preventing endemic diarrhoea?

Diarrhoea causes more than 40% of the deaths in disasters and refugee camp settings. A review identified that interventions to improve water quality at a household level are more effective than those at the source of the water [27]. This finding led to changes in policy, with the implementation of measures to safeguard the quality of water at the household level, along with the provision of safe water in emergencies. The Red Cross now includes a hygiene education component on the treatment and storage of water at the household level in the training of local volunteers in affected populations. In the decade from 2005–2015, the inclusion of this household intervention in the emergency programme is estimated to protect the health of a substantial number of people affected by disasters, perhaps as many as nine million people around the world.

**Box 4.** Systematic reviews for maternal and child nutrition interventions

*The Lancet* series on Maternal and Child Undernutrition in 2008 and 2013 are good examples of how the use of systematic reviews could help the humanitarian aid community to be informed about the effectiveness of health-related interventions. The Maternal and Child Undernutrition Series have included some high-quality systematic reviews to analyse whether the evidence for specific nutrition interventions exists or is unclear. Some Cochrane reviews suggested that vitamin A supplementation reduced all­cause mortality by 24% and diarrhoea­related mortality by 28% in children aged 6–59 months [28], intermittent iron supplementation to children younger than 2 years reduced the risk of anaemia by 49% and iron deficiency by 76% [29], and zinc supplementation in pregnancy resulted in a 14% reduction in preterm birth [30]. Reviews that suggested no or small effects of nutrition interventions included zinc supplementation in addition to antibiotics in children with severe and nonsevere pneumonia. Zinc supplementation did not have a significant effect on clinical recovery or duration of hospital stay [31], and the effectiveness of vitamin D supplementation in pregnancy revealed little evidence of benefits on functional pregnancy outcomes [32].

## Disasters: Definition and Contextual Issues

For this paper, we use the definition of the United Nations Office for Disaster Risk Reduction: “A [disaster is a] serious disruption of the functioning of a community or a society involving widespread human, material, economic or environmental losses and impacts, which exceeds the ability of the affected community or society to cope using its own resources” [5]. This definition does not differentiate between natural and man-made disasters, but from the health point of view the definition implies that a disaster is any health emergency that requires a scaled-up response through external assistance to temporarily substitute or support affected health systems.

Disasters may be the result of a sudden event such as an earthquake or a protracted cause such as malnutrition caused by famine. They may be related to epidemics or armed conflict. Often, disasters are caused by a combination of many factors, both natural and man-made, and take place in challenging political environments [6]. Health effects vary depending on the type of disaster, as well as the context (e.g., geographic, cultural, economic, political) in which it occurs (see for example the *PLOS Currents: Disasters* series on the human impact of cyclones, floods, earthquakes, tsunamis, and volcanoes [7]). For example, the burden of disease is strikingly different after earthquakes and tsunamis. Typically, earthquakes cause large numbers of injured in comparison to the numbers of dead, whereas tsunamis either kill people or leave them almost physically unscathed. If we compare Japan's 1995 earthquake with the tsunami in 2011, there was a distinct difference in the numbers of injured and dead: in 1995, there were 6.8 injured for every death, whereas in 2011 there were only 0.3 injured for every death [8].

Disasters in low-income countries are more likely to cause higher mortality and morbidity than those in middle– and high-income settings, due to a variety of reasons that include higher vulnerabilities of the population, and weaker health systems infrastructure with limited or no surge capacity. However, since the Balkan conflict in the 1990s and with the “Arab Spring,” disasters are more frequent in middle-income countries than in the past. To care for people affected by disasters in resource-limited countries, external assistance from elsewhere in the same country is often not enough and international health assistance may be needed. This “humanitarian assistance” is often guided by voluntary spirit. Whereas domestic assistance for a disaster will usually operate under defined laws and accountabilities, no such framework exists for international assistance [9]. Furthermore, there are no acknowledged professional standards or evidence-based guidelines for international health assistance [9], although the Foreign Medical Teams Working Group under the Global Health Cluster recently released its “Classification and Minimum Standards for Foreign Medical Teams in Sudden Onset Disasters” [10]. However, adherence to these standards is voluntary just as to the Sphere standards established in 1998 [11].

## Evidence for Healthcare Interventions in Disasters

Healthcare providers in disasters need readily accessible, reliable, up-to-date information on interventions that might be considered in the context of disasters. The concept of improving health through evidence-based interventions has a strong foundation in the evidence-based healthcare approach [12]. The best available evidence has been defined as the results of methodologically sound basic and patient-centred clinical research [13]. Systematic reviews of such research, including both qualitative and quantitative studies, combined with knowledge about local values, preferences, and feasibility, are needed to allow people to make well-informed decisions and choices about interventions and actions. In addition, there is a need to apply the evidence generated by patient-centred clinical research to the real world—to bridge the “know-do” gap through operational research [14].

Whereas systematic reviews are widely used for improving health in general, their role in improving health in the context of disasters is still in its infancy, but the fundamental principles of systematic reviews still apply. Systematic reviews can be used to highlight which interventions work, which do not work, which need more research, and which, no matter how well intentioned, might be harmful. In the context of systematic reviews for health interventions in disasters, it is important to remember the challenges associated with transferring evidence from one setting to another [15], and to consider the role of “realist reviews,” which seek to identify the context-mechanism-outcome, or “CMO,” configuration of interventions [16]. Also, the availability of contextual summaries and translations in different languages is important along with other means of sharing knowledge, perhaps including audio podcasts and videos.

## Our Proposal

Currently, research on disaster health interventions is scarce, as shown recently by Blanchet et al. [17]. Effort is needed to strengthen and expand the available evidence, and although randomised controlled trials may be practically difficult to conduct in disasters, other methodologies such as cohort and interrupted time-series studies could be used to address the full scope of interventions targeted at improving health in disasters. Systematic reviews for disaster health interventions need to take this general lack of a published evidence base into account. At this stage, we foresee two crucial, albeit initial, contributions that systematic reviews can make to health decision making in disasters. First, by collating and analysing the existing research, systematic reviews improve access to the available evidence for disaster health interventions and decision making. Second, systematic reviews identify knowledge gaps by showing that answers to relevant questions are not available. These knowledge gaps can then be targeted by new studies.

We use management of limb crush injuries in earthquakes as a concrete example of how systematic reviews could inform disaster health decision making. The international health response to the 2010 Haiti earthquake resulted in many calls for an evidence-based approach to limb management after crush injury. Although initial reports are likely to have given too crude a picture of the situation, Haiti was referred to as a “nation of amputees” [18]. The 2011 Humanitarian Action Summit led to a consensus statement from the surgical working group on managing limb amputations in disasters [19]. This example highlights two things. First, the performance of limb amputations after earthquakes has, up until now, been a largely subjective decision. Second, objective measures and tools are needed to guide decision making, such as outcome prediction models, but there is a lack of evidence regarding such tools in the earthquake context.

In this example, we argue that a first step would be a systematic review to identify existing tools to grade the severity and predict outcomes of crush-injured limbs. Such a review could be performed according to the Cochrane methods for a diagnostic test accuracy review [20]. The review team would ideally include both people involved in health response to disasters and researchers with experience of systematic reviews. The next step would be to assess the potential usefulness of these tools in disasters, potentially through a combination of validation studies and consensus meetings. A validation study of tools to grade injury severity could be designed as a prospective cohort study and integrated into existing systems for operational research in health response agencies. The proposed predictors would be collected along with relevant and feasible outcomes, such as mortality and functional status, at different time endpoints. In addition to its potential as a clinical decision-making aid, a tool to grade injury severity might also help with transparency and accountability in decisions about management, including amputation, helping surgeons to show how their management decisions were based on best available evidence.

## Remaining Challenges

Strengthening the evidence base to improve health care in disasters entails work on several fronts. First, a continuous dialogue is needed with the international disaster health community about the role of evidence in disasters and how best to produce and provide it. In health care, systematic reviews of randomised trials are generally considered the highest level of evidence for investigating the effects of interventions [21], but such trials can rarely be implemented in disasters due to ethical, logistical, and practical challenges [22]. Conducting research in the aftermath of disasters may be perceived as distracting from the primary objectives of saving lives and speeding recovery; however, this perception must be weighed against the need for proven and effective interventions that save the largest number of lives with the limited resources and capacities that are generally available in disasters. As research is the best way to determine which interventions are likely to be most effective, it can be argued that not conducting scientifically robust research following a disaster is unethical [23]. Second, existing systematic reviews need to be identified and made available in a free, easily accessible format. Third, effective knowledge transfer is needed to help the international disaster health community to identify, conduct, and use research, including systematic reviews. Fourth, better understanding is needed of how people make decisions about interventions—how they combine the best available evidence with contextual, cultural, organisational, and stakeholder issues—and the optimal ways of doing this. Fifth, funding needs to be ensured through special grants, such as the Enhancing Learning and Research for Humanitarian Assistance (ELRHA) Rapid Response Grant [24].

In conclusion, there needs to be a paradigm shift in healthcare decision making in disaster preparedness and response, moving towards a reliable and robust evidence base for all interventions being considered in disaster risk reduction, planning, response, and recovery. Evidence Aid presents an opportunity for all those involved in disaster response to collaborate in developing and enacting the best available evidence, so as to ensure that they have the best knowledge needed to decide how to respond in the worst of times.
